# Hereditary Systemic Autoinflammatory Diseases: Therapeutic Stratification

**DOI:** 10.3389/fped.2022.867679

**Published:** 2022-04-28

**Authors:** Ovgu Kul Cinar, Amber Putland, Karen Wynne, Despina Eleftheriou, Paul A. Brogan

**Affiliations:** ^1^Department of Paediatric Rheumatology, Great Ormond Street Hospital for Children NHS Foundation Trust, London, United Kingdom; ^2^Division of Medicine, National Amyloidosis Centre and Centre for Acute Phase Proteins, University College London, Royal Free Campus, London, United Kingdom; ^3^Section of Infection, Immunity and Inflammation, Institute of Child Health, University College London Great Ormond Street, London, United Kingdom; ^4^Paediatric Rheumatology, ARUK Centre for Adolescent Rheumatology, Institute of Child Health, University College London (UCL) Great Ormond Street Hospital, London, United Kingdom

**Keywords:** genomics, IL-1 inhibitors, innate immunity, interferonopathies, JAK 1/2 inhibitors, periodic fever, systemic autoinflammation

## Abstract

Hereditary systemic autoinflammatory diseases (SAIDs) are rare, often severe conditions characterised by mutations in the key regulators of innate immune responses. Dramatic advances in the molecular genetics and next-generation sequencing in the past decade enabled identification of novel mutations that play a pivotal role in the mechanistic pathways of inflammation. Although genetic testing may not always provide straightforward guidance in diagnosis and clinical decision making, through translational research, it sheds light into molecular immunopathogenesis, particularly in IL-1 inflammasome and cytokine signalling pathways. These remarkable insights provided a better understanding of autoinflammatory conditions and their association with the innate and adaptive immune systems, as well as leading to development of cytokine-targetted biologic treatments. Use of targetted therapeutics not only helps control disease flares, reduce acute-phase responses and prevent devastating complications such as amyloidosis, but also improves health-related quality of lives and support patients to pursue almost a normal life. Herein, we discuss the commonest monogenic SAIDs, describe their immunopathology, and summarise the approaches in the management and targetted treatment of these conditions, including presentation of novel data based on a cohort of children with these rare diseases from a single quaternary referral centre in London.

## Introduction

The concept of systemic autoinflammatory diseases (SAIDs) was first proposed by McDermott et al. in 1999, as a group of hereditary conditions characterised by recurrent unprovoked inflammation, without presence of autoantibodies or antigen-specific T-lymphocytes (1). Abundant inflammatory response is predominantly mediated by the cells and molecules of the innate immune system in the presence of host predisposition (1). The autoinflammatory nomenclature sought to provide a unified classification for this ever-growing list of diseases that can be distinguished from autoimmune conditions, without major involvement of adaptive immune system which is the hallmark for autoimmunity (2).

Systemic autoinflammatory diseases (SAIDs) usually present with unexplained recurrent episodes of fever and multisystem inflammation; mainly involving serosal surfaces, synovium, skin and eyes (3). Inflammation in muscles, vasculitis affecting small, medium and large vessels, and systemic amyloidosis may also occur in some patients (4). Hereditary recurrent fevers (HRFs) constitute a subgroup of SAIDs which involves familial Mediterranean fever (FMF), tumour necrosis factor receptor-associated periodic syndrome (TRAPS), cryopyrin-associated periodic fever syndrome (CAPS) and hyperimmunoglobulin-D with periodic fever syndrome (HIDS, now referred to as mevalonate kinase deficiency, MKD) as prototypes for this diagnostic category (2). Nevertheless, in addition to HRFs, the concept of autoinflammation has now been extended to a number of clinical entities encompassing more recently identified Mendelian diseases such as deficiency of adenosine deaminase-2 (DADA-2) and monogenic interferonopathies; as well as diseases with a polygenic form of inheritance (such as Behçet’s and Still’s disease) (2).

Advances in molecular genetics and the increased accessibility to genetic testing have extended recognition and treatment of SAIDs to a wider range of populations and ethnicities, which in turn provided important insights into genotype-phenotype associations (5). Diagnosis of SAIDs relies fundamentally on good history taking with a detailed family history, and clinical judgement. Molecular genetic analyses provide a definitive diagnosis for many cases, although results require careful interpretation as they can be misleading or inconclusive especially in the context of variants of uncertain significance (VUS) (6, 7). Over the last two decades, at least 30 different genes have been identified in hereditary diseases (Infevers^1^); as well as detailed descriptions of a growing number of polygenic “complex” syndromes (3). Recognition of underlying mutations in monogenic SAIDs has led to the identification of key regulators of innate immune responses. The innate immune system with its myeloid effector cells and pattern-recognition receptors (PRRs) for pathogen and danger-associated molecular patterns drives the immune responses in SAIDs (2, 8). NOD-like receptors (NLRs) that are a group of PRRs in innate immunity, have been identified as intracellular sensors with an ability to sense non-microbial danger signals, and subsequently form large cytoplasmic inflammasomes that activates caspases and results in secretion of pro-inflammatory cytokines IL-1ß and IL-18 (2, 9, 10). Recognition of the disease-associated mutations in the genes encoding inflammasome pathway, as an example mutations in *MEFV*, *NLRP3*, and *TNFRSF1A*, has dramatically improved our understanding of disease pathogenesis, and thereby had a vital impact on the development of targetted-therapeutic approaches (11). Excessive IL-1ß production and IL-1 signalling as a consequence of inflammasome assembly and activation upon *NLRP3* stimulation in CAPS spectrum represents an exemplar of targetted-therapeutic approach based on selective cytokine blockade to control inflammation (11). Similarly, “pyrin” protein encoded by the *MEFV* gene is linked with cytoskeleton in myeloid/monocytic cells and modulates IL-1ß processing, nuclear factor NF-κB activation and apoptosis which can explain the hyperinflammatory state during FMF attacks (12).

The underlying mechanisms of SAIDs is now better elucidated and it has been clarified that autoinflammation can be triggered in the context of activation of various inflammatory pathways, cytokine signalling or accumulation of misfolding proteins. In addition to above-described inflammasomopathies in FMF and CAPS, other underlying mechanisms for SAIDs can be summarised as follows: intracellular stress that causes production of reactive oxygen species; aberrant apoptosis; protein-misfolding and aberrant cytokine production as in TRAPS and HIDS; increased interferon signalling as in SAVI and CANDLE; NF-κB activation disorder as in Blau syndrome; and deficiency of enzymes such as adenosine deaminase 2 causing autoinflammation and vasculitis (2, 3) (Table 1).

**TABLE 1:** Monogenic systemic autoinflammatory diseases.

Disease	Gene/chromosome	Protein	Mode of inheritance
**IL-1ß activation disorders (inflammasomopathies)**			
FMF	*MEFV* (16p13.3)	Pyrin (marenostrin)	Autosomal recessive/or gene-dosage dependent autosomal dominant
CAPS [FCAS/MWS/CINCA (NOMID)]	*NLRP3* (1q44)	NLRP3 (prev. cryopyrin, NALP3)	Autosomal dominant
PAPA	*PSTPIP1* (15q24-25.1)	PSTPIP1	Autosomal dominant
AIFEC	*NLRC4 (2p22.3)*	NLRC4	Autosomal dominant
**Intracellular stress leading to inflammation (protein misfolding, dysregulated ubiquitination, abnormal intracellular accumulation and intracellular trafficking of mutant proteins)**			
TRAPS	*TNFRSF1A (12p13.31)*	TNFR1	Autosomal dominant
MKD/HIDS	*MVK (12q24.11)*	Mevalonate kinase	Autosomal recessive
Haploinsufficiency A20	*TNFAIP3 (6q23.3)*	TNFAIP3	Autosomal dominant
PFIT	*WDR1 (4p16.1)*	WDR1	Autosomal recessive
VEXAS	*UBA1 (Xp11.3)*	UBA1	X-linked
**Defective regulatory mechanisms (affecting cytokine signalling)**			
DIRA	*IL1RN (2q14.1)*	IL-1Ra	Autosomal recessive
DITRA	*IL36RN (2q14.1)*	IL-36Ra	Autosomal recessive
Majeed syndrome	*LPIN2* (18p11.31)	Lipin-2	Autosomal recessive
**Defects in NF-κ B signalling pathway**			
Blau syndrome	*NOD2 (CARD15) (16p12)*	NOD2 (CARD15)	Autosomal dominant
NEMO-NDAS	*IKBKG (Xq28)*	IKBKG	X-linked
**ADA-2 Deficiency**			
DADA-2	*ADA-2 (prev. CECR1) (22q11.1)*	ADA-2	Autosomal recessive
Increased intracellular Ca^+2^ signalling			
APLAID	*PLCG2 (16q23.3)*	PLCG2	Autosomal dominant
**Interferonopathies**			
CANDLE/PRAAS	*PSMB8 (6p21.32) PSMB4 (1q21.3) PSMA3 (14q23.1) PSMB9 (6p21.32) PSMB10 (16q22.1) PSMG2 (18p11.21) POMP (13q12.3)*	PSMB8, PSMB4, PSMA3, PSMB9, POMP	Autosomal recessive
SAVI	*TMEM173 (STING1) (5q31.2)*	STING1	Autosomal dominant
AGS	*TREX1(3p21.31) IFIH1 (2q24.2) SAMHD1 (20q11.23) RNASEH2A-C (19p13.13, 13q14.3, 11q13.1) ADAR (1q21.3) RNU7-1 (12p13.31) LSM11 (5q33.3)*	TREX1 IFIH1 SAMHD1 RNASEH2A-C DRADA	Autosomal dominant and autosomal recessive

*ADA-2, Adenosine deaminase-2; ADAR, Adenosine deaminase RNA specific; AGS, Aicardi-Goutières Syndrome; AIFEC, autoinflammation and infantile enterocolitis; APLAID, PLCG2-associated antibody deficiency and immune dysregulation with autoinflammation; CANDLE, Chronic Atypical Neutrophilic Dermatosis with Lipodystrophy and Elevated temperature; CAPS, Cryopyrin-associated periodic syndrome; CARD15, caspase recruitment domain-containing protein 15; CECR1, cat eye syndrome critical region protein 1; CINCA, Chronic infantile neurological cutaneous and articular syndrome; DADA-2, deficiency of adenosine deaminase-2; DRADA; Double stranded RNA binding protein; FCAS, Familial cold autoinflammatory syndrome; FMF, familial Mediterranean fever; HIDS, hyperimmunoglobulin D syndrome; IFIH1, Interferon induced with helicase C domain 1; IKBKG, inhibitor of nuclear factor kappa B kinase regulatory subunit gamma; IL-1ß, interleukin 1 beta; IL1RN, interleukin 1 receptor antagonist; IL36RN, interleukin 36 receptor antagonist; LPIN2, lipin 2; LSM11, U7 Small nuclear RNA-associated protein; MEFV, MEditerranean Fever; MKD, mevalonate kinase deficiency; MVK, mevalonate kinase; MWS, Muckle-Wells syndrome; NEMO-NDAS, NF-κB essential modulator delta exon 5-autoinflammatory syndrome; NF-κB, nuclear factor kappa B; NLRC4, NLR-family CARD domain containing protein 4; NLRP3, NOD, LRR and pyrin domain-containing protein; NOD2, nucleotide binding oligomerisation domain containing protein 2; NOMID, neonatal-onset multisystem inflammatory disease; PAPA, pyogenic arthritis, pyoderma gangrenosum, and acne; PFIT, periodic fevers immunodeficiency and thrombocytopaenia; PLCG2, phosphatidylinositol-specific phospholipase C gamma 2; POMP, Proteasome maturation protein; PSMA3, Proteasome 20S subunit alpha 3; PSMB 4/8/9, Proteasome 20S subunit beta 4/8/9; PSMG2, proteasome assembly chaperone 2; PSTPIP1, proline-serine-threonine phosphatase interacting protein 1; RNASE H1A-C, ribonuclease H1A-C; RNU7-1, RNA, U7 Small Nuclear 1, SAMHD1, SAM and HD domain containing deoxynucleoside triphosphate triphosphohydrolase 1; SAVI, STING-associated vasculopathy of infancy; STING1, stimulator of interferon response CGAMP interactor 1; TMEM173, transmembrane protein 173; TNFAIP3, TNF alpha induced protein 3; TNFR1, tumour necrosis factor receptor 1; TNFRSF1A, TNF receptor superfamily member 1A; TRAPS, tumour necrosis factor receptor-associated periodic syndrome; TREX1, three prime repair exonuclease 1; UBA1, ubiquitin like modifier activating enzyme 1; VEXAS (vacuoles, E1 enzyme, X-linked, autoinflammatory, somatic) syndrome; WDR1, WD repeat domain 1.*

Recently discovered inherited SAIDs such as the Behcet’s mimic haploinsufficiency of A20 (13), VEXAS (vacuoles, E1 enzyme, X-linked, autoinflammatory, somatic) syndrome (14), autoinflammation with infantile enterocolitis (AIFEC) (15), periodic fevers, immunodeficiency and thrombocytopenia (PFIT) (16) and NEMO 5-associated autoinflammatory syndrome (NEMO-NDAS) (17) (Table 1) have further expanded the knowledge on immunobiology of innate immunity and autoinflammation, which in turn provided important insights for the treatment of these conditions.

This review summarises the immunopathological and clinical features of SAIDs with the most recent advances in the targetted-therapeutic approaches in line with the discoveries in basic and translational science. It will also outline the caveats in the management and treatment of SAIDs and finally, will point out the importance of precision medicine in SAIDs with an emphasis on how molecular insights can form the conceptual basis for targetted treatment within a real-life clinical framework.

## Familial Mediterranean Fever

Familial Mediterranean Fever (FMF) is the most prevalent and well-described hereditary SAID primarily affecting ethnic groups originating from eastern Mediterranean region, such as non-Ashkenazi Jews, Greeks, Turks, Armenians and Arabs, though it is now increasingly recognised in different populations owing to enhanced accessibility to genetic testing and raised disease awareness amongst physicians (18, 19).

The gene responsible for FMF, *MEFV* (MEditerranean Fever), is composed of 10 exons on chromosome 16p13.3 and encodes pyrin (or marenostrin) protein which is a component of IL-1 inflammasome pathway, hence regulates IL-1ß processing and activation (20, 21). According to INFEVERS database, more than 380 *MEFV* sequence variants have hitherto been reported^2^ some of which are clearly pathogenic, whereas a large number of variants currently remain as of unknown significance (22). FMF is traditionally known as an autosomal recessive disease, nevertheless substantial number of patients with clinical FMF phenotype demonstrate only 1 identifiable *MEFV* mutation. To investigate this further, Booty *et al.* searched the existence of a second mutation in patients clinically diagnosed with FMF and revealed that none of the patients had a second mutation (23). This study has demonstrated that FMF may not be a simple monogenic inflammatory disease and patients with only 1 *MEFV* mutation can present with FMF phenotype in the presence of other permissive alleles or environmental factors (23). Similarly, Marek-Yagel et al. investigated heterozygote FMF patients and performed haplotype analyses which revealed that heterozygote disease was indistinguishable from homozygote form, thus FMF can be considered as a dominant condition with low penetrance (24). The Single Hub and Access point for pediatric Rheumatology in Europe (SHARE) initiative in 2015 recommended that the presence of homozygous mutations in p.M694V, p.M680I, p.M94I or compound heterozygous mutations with two of these genes were associated with increased risk of severe disease (22). In contrast, although very common, presence of the p.E148Q variant as the only *MEFV* variant was recommended not to be labelled as FMF (evidence strength level B) (22). These recommendations were also demonstrated in the meta-analysis by Gangemi et al. (7) supporting the evidence regarding the diagnosis of FMF being based on clinical criteria, supported with genetic testing. Booty et al. (23) showed that almost 30% of FMF patients with clinical symptoms had only one identifiable mutation in *MEFV*. Thus it is suggested that FMF is best described an autosomal dominant condition with variable penetrance, and with gene mutation dosing effect influencing phenotypic severity. The variability of attack presentation and symptoms is currently explained by the effect of different MEFV mutations and compound heterozygotes, as well as by the contribution of heterogeneity among disease-modifying proteins (25). In 2012, experts reached consensus on performing genetic test for the 14 most common MEFV variants, nine of which are clearly pathogenic and five are VUS (6).

Familial Mediterranean fever (FMF) presents with self-limited inflammatory episodes of fever accompanied by polyserositis, arthritis and/or an erysipelas-like skin disease, all of which spontaneously resolve in 48 to 72 h (18). One of the potential underlying mechanisms is a massive increase in the chemotactic activity of the polymorphonuclear leucocytes resulting in rapid granulocyte influx into the affected tissues, driving autoinflammation (18). Increased understanding of innate immune system responses to different pathogens and pattern recognition receptors (PRRs) have led to the elucidation of potential immune mechanisms: pyrin inflammasome activation was triggered by the inhibition of RhoA, a member of the Ras homology family of small GTPases (26). In line with this hypothesis, following inactivation of RhoA by *Clostridium difficile* virulence factor, cytotoxin TcdB, pyrin mediated caspase 1-inflammasome activation (26). Indeed, Xu et al. demonstrated robust activation of caspase-1 and IL-1ß production by a recombinant Clostridium cytotoxin TcdB. This hypothesis was later underpinned by Park et al. (27) demonstrating pyrin inflammasome upon inhibition of Rho GTPases by certain bacterial toxins. Unlike other pathogens, *Yersinia* species were shown to possess a specific human pyrin inflammasome inhibiting toxin, YopM which facilitated binding of inhibitory proteins to the pyrin (28). IL-1ß release was substantially reduced in wild-type *Yersinia pestis* infected bone-marrow derived macrophages, whereas FMF patients with mutated pyrin could effectively produce IL-1ß upon being infected with *Y. pestis* (29). Park et al. (29), then demonstrated that YopM binding to FMF mutant human pyrin was markedly decreased compared to binding of toxin to the wild type human pyrin. Moreover, levels of IL-1ß secretion in the presence of different FMF-associated mutations and wild-type pyrin were measured. Interestingly, the cells expressing classical pathogenic (*MEFV* p.M694V, p.M680I, p.V726A) FMF mutations, hence FMF-associated mutant pyrin, secreted significantly higher levels of IL-1ß in comparison to the cells expressing wild-type pyrin or those with the *MEFV* p.E148Q variant (29). Taken together, these studies provided valuable knowledge about FMF and inflammasome pathophysiology, and opened avenues for future research.

In terms of morbidity, systemic AA amyloidosis is the most severe complication of FMF that generally presents with renal involvement (11%), though the adrenals, spleen, intestine, lung, and testes can also be affected (30, 31). Of note, AA amyloidosis used to cause long-term morbidity and mortality in majority of the patients before discovery of colchicine. Indeed, colchicine has been successfully used as a first line medication in FMF since 1972 not only for controlling attacks but also for preventing and treating amyloidosis (30, 32). Interestingly, in the abovementioned study by Park et al. (27), colchicine was shown to activate RhoA or reverse inhibition of it by depolymerisation of intracellular microtubules, and ultimately inhibited inflammasome and IL-1ß release. Recently, evidence-based recommendations for the management of FMF have been published by European League Against Rheumatism (EULAR) with international collaboration (33), which suggested prompt start of prophylactic colchicine as soon as the clinical FMF diagnosis is confirmed. Notably, in the absence of clinical FMF diagnosis or subclinical inflammation, genetic diagnosis is not a prerequisite to start treatment, although regular monitoring of these patients was recommended (33).

It is important to emphasise that colchicine is a safe and well-tolerated life-long treatment for the prophylaxis of FMF attacks, the commonest side effect being gastrointestinal disturbance with diarrhoea occurring predominantly in the first month of treatment (19). Dose reduction and a lactose-free diet may alleviate side effects, as colchicine is considered to unmask lactose intolerance (34). It has been reported that up to 5% of patients may remain colchicine intolerant, and in 5-10% disease is only partially controlled with colchicine (35). EULAR recommendations highlight that reduced compliance should be seriously considered in apparent colchicine resistant FMF patients (33). The underlying aetiology for intolerance has not been completely clarified yet; however, it is likely to be multifactorial, potentially associated with genetic variants and pharmacokinetics such as absorption and intracellular transport as well as interaction with other medications (36, 37). Clinicians should be vigilant whilst assessing patients, especially those with increased attack frequency and/or severity or colchicine unresponsiveness (19) as treatment adherence can be a major problem for some cases, particularly during adolescence. Colchicine-intolerant or inadequately controlled cases or those with hepatobiliary dysfunction and/or severe renal failure are candidates for alternative treatment options, namely IL-1 inhibitors (38).

Given the roles of mutated pyrin protein in regulating caspase-1 and IL-1ß activation in inflammasome pathway, recent studies have demonstrated successful use of anti-IL1 therapies for the treatment of FMF (21, 25, 39, 40). To date, there are three different anti-IL1 medications effectively used in clinical practice (19): anakinra, a recombinant homolog of the human IL-1 receptor antagonist, competitively blocks binding of IL-1alpha and IL-1ß to the IL-1 receptor. Canakinumab, a fully human immunoglobulin G_1_ monoclonal antibody against IL-1ß and rilonacept, a dimeric fusion protein capturing IL-1 (19, 25).

Non-steroidal anti-inflammatory drugs (NSAIDs) can be helpful as on-demand therapy to control symptoms during attacks, although they are not promising in preventing further episodes. The only indication for corticosteroid use in FMF is protracted febrile myalgia which is a very rare vasculitic complication (41).

## Tumour Necrosis Factor Receptor-Associated Periodic Syndrome

Tumour Necrosis Factor Receptor-Associated Periodic Syndrome (TRAPS) is a monogenic AID caused by mutations in the extracellular domain of the TNF receptor (*TNFRSF1A*) on chromosome 12 which is recognised to have a central role in the regulation of innate immune responses and inflammation (4). The biologic effects of TNF include expression of adhesion molecules on leucocytes and endothelial cell surfaces, stimulation of cytokine secretion, leucocyte activation, angiogenesis and pyrexia (4). It was initially thought that mutations in type 1 TNF receptor (TNFR1) caused reduction in well-functioning soluble receptors leading to excess circulating TNF that would fuel inflammatory responses (11). Based on this pathophysiological mechanism, the TNF-inhibitor etanercept seemed to be the best possible therapeutic option at that stage. However, lack of efficacy of etanercept resulted in questions regarding the true pathogenesis of TRAPS (42). Defective clearance and intracellular trafficking of TNFR1 ultimately leads to accumulation of mutant receptor in endoplasmic reticulum (ER), potentially increasing intracellular stress and production of reactive oxygen species by mitochondria (43). Interaction between ER stress, ROS production and IL-1ß secretion has been established not only in TRAPS but also for other autoinflammatory diseases; moreover, this association might be a plausible explanation for the effectiveness of IL-1 inhibition in TRAPS (44). Indeed, initial anecdotal reports on promising use of anti-IL1 agents have been followed by the Eurofever Registry data (45), international expert consensus reports (46) and eventually a placebo-controlled, randomised study of canakinumab (39) proving IL-1 blockade as a safe and effective therapy for TRAPS, emphasising the pivotal role of IL-1 in the pathogenesis.

Regarding the presenting symptoms during attacks, alongside episodic fever, serositis and arthritis, patients also experience conjunctival involvement and/or periorbital oedema and myalgia. Another feature of this periodic fever syndrome is longer attack duration as compared with other hereditary SAIDs. Many TRAPS episodes last from less than one week to up to three weeks and minority of patients may have continuous inflammation with exacerbations (3, 4).

Evidence for treatment of TRAPS attacks is limited to retrospective or small prospective studies (46). NSAIDs have been shown to provide symptom relief in approximately 75% of TRAPS patients, although they are not effective in controlling inflammatory episodes. Likewise, corticosteroid effect was assessed in only retrospective studies and favourable outcomes were reported as on-demand therapy, nonetheless, initial response reduced over time, in addition to corticosteroid related side effects (47). In the Eurofever Registry, patients with the mild R92Q variant seemed to respond better to NSAIDs and colchicine compared to the other TNFRSF1A mutations, though overall colchicine use was not found beneficial (45). In prospective and retrospective studies, TNF-inhibitor etanercept demonstrated favourable outcomes accompanied with reduction in inflammatory parameters; nevertheless, efficacy declined over time (45, 46). Treatment failure, and even deterioration was observed with monoclonal TNF-inhibitors – infliximab and adalimumab, thus use of these medications are no longer recommended (48). In the Eurofever Registry (45) anti-IL1 treatment with anakinra was shown to be superior to etanercept, and indeed, recently an open-label canakinumab study (49) and placebo-controlled trial (39) confirmed efficacy of anti-IL1 therapies in TRAPS which led to licencing of canakinumab by U.S. Food and Drug Administration and European Medicines Agency.

## Cryopyrin-Associated Periodic Syndrome

**FIGURE 1:**
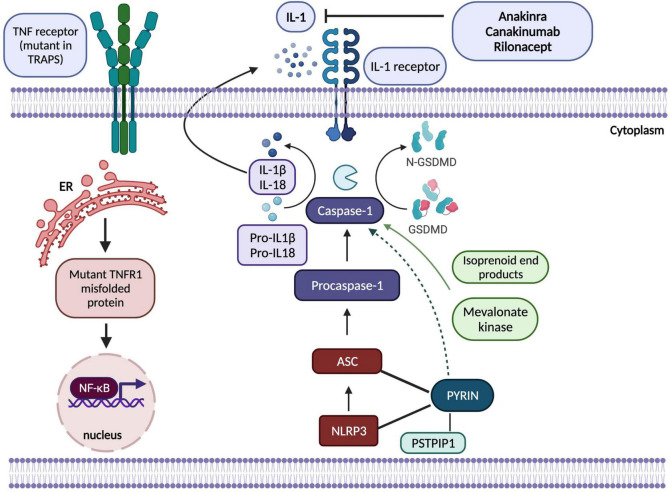
Adapted from Kastner et al. (2) a schematic showing pathogenesis of hereditary autoinflammatory diseases regulated by IL-1ß (inflammasomopathies) and NF-κB (Created by Biorender). In CAPS, NLRP3 protein interacts with the adaptor protein ASC and caspase-1 to form the inflammasome complex which activates IL-1ß and IL-18 and results in inflammatory responses. Mutations in pyrin, PSTPIP1, NLRP3, mevalonate kinase proteins shown in the figure disrupt normal functioning of inflammasome complex. Mutations in NLRP3 increase the activation of the complex; however, mechanisms by which pyrin protein regulates inflammasome functioning have yet to be elucidated as theories involve both gain-of-function activation of pyrin-inflammasome complex and loss-of-function in its inhibitory effects on NLRP3. In TRAPS, mutant TNFR1 protein is misfolded in ER that causes accumulation of this protein, hence overactivation of NF-κB resulting in abnormal inflammatory immune responses. IL-1-targetted biologic agents, anakinra, canakinumab and rilonacept have been effectively used to control inflammatory state in these prototypic monogenic SAIDs. ASC, Apoptosis-associated speck-like protein; ER, endoplasmic reticulum; GSDMD, Gasdermin D; IL-1, interleukin-1; NF-κB, nuclear factor κB; NLRP3, NOD, LRR and pyrin domain-containing protein; PSTPIP1, proline-serine-threonine phosphatase interacting protein 1; TNF, tumour necrosis factor, TNFR1, tumour necrosis factor receptor 1; TRAPS, TNF-receptor associated periodic syndrome.

Cryopyrin-associated periodic syndrome (CAPS) is a rare, heterogenous inflammasomopathy associated with gain-of-function or *de novo* mutations in *NLRP3* that encodes a protein which is known as *NLRP3* or cryopyrin, a regulatory protein in interleukin-1 inflammasome (50) (Figure 1). Mutations in *NLRP3* result in excessive IL-1ß production causing a wide range of symptoms from cold-induced urticaria to severe neurological inflammation. Indeed, the clinical spectrum of CAPS is broad and comprises distinct and rare subgroups in regards to symptom severity (51). From mild to severe: familial cold autoinflammatory syndrome which can present with cold-induced urticaria, fever and constitutional symptoms; Muckle-Wells Syndrome (MWS) with temperatures, skin rashes, arthritis and sensorineural hearing loss; and the most severe form being chronic infantile neurological cutaneous articular syndrome (CINCA) [or neonatal-onset multisystem inflammatory disease (NOMID)] which can present with temperature episodes, arthritis or myalgia, cutaneous symptoms, chronic aseptic meningitis and epiphyseal overgrowth of the long bones (51). In the light of *NLRP3* being a component of inflammasome which senses danger signals and activates caspase-1, thereby initiating IL-1ß and IL-18 processing, IL-1 blockade resulted in complete therapeutic responses which have been life-altering in this monogenic inflammasomopathy (45, 50). Long-term IL-1 inhibition is now indicated for the whole spectrum of CAPS at any age, and the consensus recommendation is to initiate treatment as early in life as possible (46). In addition to IL-1 blockade, use of corticosteroids and/or non-steroidal anti-inflammatory drugs (NSAIDs) as on-demand therapy during attacks has also proven beneficial for some patients (46).

Systemic AA amyloidosis, a life-threatening complication of SAIDs that mainly presents with renal involvement, was reported to occur in approximately 25% of CAPS patients prior to targetted treatment with IL1 blockade (52). In a double-blind, place-controlled study, treatment with canakinumab demonstrated a rapid and sustained clinical response in CAPS patients with a substantial reduction in acute-phase reactants, especially in serum amyloid A, indicative of reduced AA amyloidosis risk (52). Canakinumab was considered as superior to other IL-1 inhibitors for multiple reasons. Firstly, subcutaneous administration of canakinumab is every four to eight weeks, whereas other IL-1 inhibitors anakinra and rilonacept are given daily or weekly, respectively. Undoubtedly, less frequent injections will increase adherence among adolescent and adult patients. Secondly, longer plasma half-life up to 28-30 days presumably provides prolonged resolution of symptoms and autocrine downregulation of IL-1ß production may be another beneficial disease-modifying impact (52). And finally, in the placebo-controlled, double-blind trial, at the end of week 24, all canakinumab patients remained in remission as compared with 25% in the placebo arm (52).

## Mevalonate Kinase Deficiency or Hyperimmunoglobulin D With Periodic Fever Syndrome

Mevalonate Kinase Deficiency (MKD)/Hyperimmunoglobulin D With Periodic Fever Syndrome (HIDS) (OMIM #260920) is a rare autosomal recessive SAID characterised by bi-allelic mutations in the mevalonate kinase (*MVK)* gene (12q24.11) resulting in defective cholesterol biosynthesis and increased urinary excretion of mevalonic acid (53). The disease spectrum is primarily a continuum of two clinical phenotypes with different severity: at the milder end, MKD/HIDS sits as an autoinflammatory condition with early-onset febrile episodes usually without infection, skin rash, arthralgia, hepatosplenomegaly, lymphadenopathies, gastrointestinal involvement, but absence of neurological impairment. The metabolic disease mevalonic aciduria (MVA) remains at the severe end of the spectrum. MVA presents early in life with similar features, but later on the clinical phenotype includes neurodevelopmental delay, muscular hypotonia, ataxia associated with cerebellar atrophy and ocular symptoms (53). Both conditions are caused by homozygous or compound heterozygous mutations in *MVK* (53). The enzymatic activity of MVK can be measured in fibroblasts and lymphoblasts and varies from 1.8 to 28% in the autoinflammatory form; whereas it is usually below 0.5% in MVA (53, 54).

Symptom onset is usually in the first 6 months of life with recurrent 3- to 7-day temperature episodes that can be precipitated by specific factors such as vaccination, infections, surgery, physical or emotional stress. Macrophage activation syndrome (0.9%) and AA amyloidosis (4%) are rare but severe complications (54).

The aetiopathogenesis of autoinflammation in MKD has not been fully elucidated yet, although recent studies emphasise the role of defective prenylation in RhoA inactivation (27). Mevalonate kinase (MVK) is a central enzyme catalysing the isoprenoid biosynthesis pathway, generating sterol and non-sterol isoprenoids utilised in essential cellular functions (55). Autoinflammation and pyrin inflammasome activation in MKD has indicated the role of isoprenoids in the regulatory innate immune mechanisms (55). Downstream molecules in cholesterol biosynthesis such as geranylgeranyl pyrophosphate (GGPP) were shown to be used in protein prenylation by post-translational attachment of these molecules to the target proteins for optimum membrane localisation (55, 56). As an explanatory mechanism, Park et al. (27) demonstrated that membrane targetting of RhoA was also dependent on geranylgeranylation, which was compromised in the absence of GGPP resulting in RhoA inactivation and consequent pyrin inflammasome activation.

Treatment of MKD has been challenging and different medications have been trialled. Colchicine and statins were not effective in the majority of patients (54, 57). NSAIDs and corticosteroids have been used with some benefit for symptomatic relief, but neither of them was completely efficacious (54).

Maintenance therapy with IL-1 blockade and etanercept has been recommended in the international consensus guidance (46), although therapeutic success with daily anakinra is generally limited, with only 22% complete remission; and 89% partial remission (45). In an open-label study, Arostegui *et al.* (58) demonstrated that canakinumab was an effective treatment choice to control MKD attacks as well as to suppress inflammation-related transcriptional responses (58). More recently, a placebo-controlled trial has also shown favourable outcomes with canakinumab treatment which led licencing of this medication in MKD (39).

## Deficiency of Adenosine Deaminase-2

Deficiency of Adenosine Deaminase-2 (DADA-2) is a recently described autosomal recessive SAID caused by loss-of-function homozygous or compound heterozygous mutations in adenosine deaminase-2 *(ADA-2)* (previously *CECR1)* (59). Navon Elkan et al. (60) first identified mutations in this gene by whole exome sequencing (WES) in families with polyarteritis nodosa (PAN) (60). Zhou et al. (59) reported that skin, liver and brain biopsies revealed vasculopathic changes with compromised endothelial integrity leading to endothelial cellular activation and inflammation (59). ADA-2 enzyme is produced by myeloid cells and promotes differentiation of monocytes and anti-inflammatory M2 macrophages, hence deficiency of ADA-2 results in a predominance of pro-inflammatory M1 macrophages that secrete pro-inflammatory cytokines, mainly TNFα (59, 61).

**FIGURE 2:**
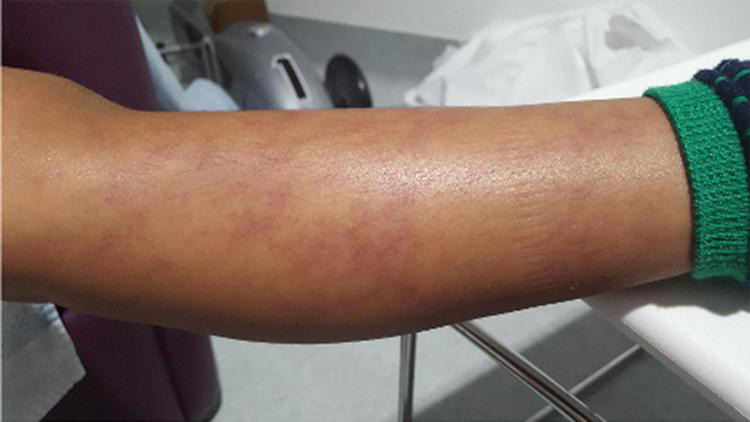
Livedo racemosa type vasculitic skin rash in a DADA-2 patient. DADA-2, Deficiency of adenosine deaminase-2.

The clinical and histopathological features are very similar to PAN, and characterised by systemic inflammation, livedo racemosa (Figure 2), and early-onset vasculopathy which may cause haemorrhagic and ischaemic lacunar strokes (62). Multifaceted phenotypes with immunodeficiency and/or haematological involvement ranging from pure red-cell aplasia (Diamond-Blackfan anaemia) and neutropaenia to multi-lineage bone marrow failure have been reported (63, 64).

Definitive diagnosis depends on mutation detection and demonstration of reduced ADA2 enzyme activity in serum (61). Retrospective cohort studies have demonstrated profound therapeutic efficacy and safety of TNF-inhibitors which provided complete control of inflammation, reduced vasculitic disease activity and prevented occurrence of vascular events without causing severe complications (61, 62, 65). Infusion reactions to infliximab (human-mouse chimeric monoclonal antibody) that require switching to another anti-TNF agent (etanercept or adalimumab) have been reported (61). Given that DADA-2 patients require lifelong treatment, development of anti-drug antibodies especially against monoclonal antibodies and reduction in treatment efficacy has been a major concern for anti-TNF therapies. Furthermore, TNF-inhibitors have not been beneficial in treating DADA-2 patients with haematological manifestations or immunodeficiencies, although allogeneic haematopoietic stem cell transplantation has been proven successful to control these symptoms as well as vascular inflammation (64–66). Aspirin usage has been debatable in DADA-2; however, in some cases with non-haemorrhagic ischaemia and peripheral vascular disease, judicious use of aspirin to obtain acute anti-thrombotic effect may be beneficial (61, 67). Gene therapy is currently being studied and is a potential future therapeutic option for DADA-2 patients as it has been promising in other monogenic diseases (61).

## Interferonopathies

Interferon (IFN)-mediated systemic autoinflammatory diseases are innate immune dysregulatory diseases that present early in life with fever, systemic inflammation, neuroinflammation, and upregulated type I IFN response gene signatures (ISGs) in peripheral blood cells. They usually have high morbidity and mortality rates (68, 69). Monogenic interferonopathies include *C*hronic *A*typical *N*eutrophilic *D*ermatosis with *L*ipodystrophy and *E*levated temperature (CANDLE), STING-associated vasculopathy of infancy (SAVI), and Aicardi-Goutières Syndrome (AGS) (68). CANDLE [also referred as proteasome-associated autoinflammatory syndrome (PRAAS)] is caused by loss-of-function mutations in genes encoding proteasome-immunoproteasome components that regulate protein degradation. Brehm et al. (70) revealed that mutations in proteasome genes had an impact on protein expression, protein folding, proteasome assembly and activity. The outcome is sustained IFN-gene expression signature, regardless of genotype. Likewise, SAVI is known to be caused by gain-of-function mutations in *STING1*, the gene encoding stimulator of interferon genes (STING) which plays a central role in innate immune responses by stimulating type I IFN production (71). Disease pathogenesis is similar and considered to be driven by the elevated ISGs; thus, these conditions present with overlapping clinical phenotypes (71).

**FIGURE 3:**
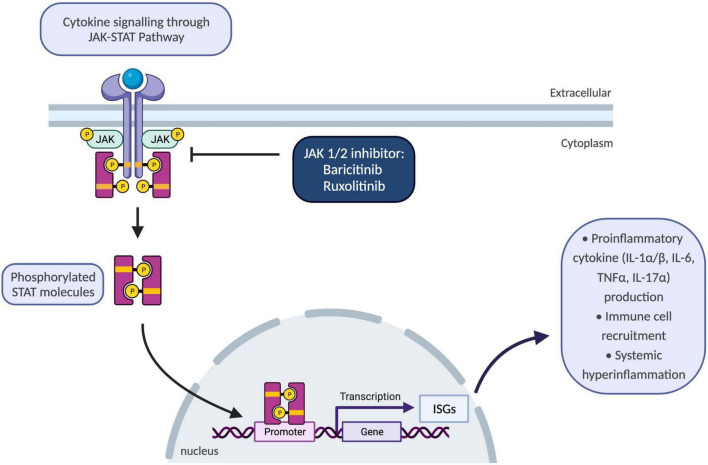
Schematic showing JAK-STAT pathway and the use of JAK 1/2 inhibitors to block inflammatory responses. JAK/STAT pathway plays a key role for the downstream signalling of inflammatory cytokines encompassing interleukins and interferons. Upon binding of cytokines to their cognate receptors on cell surface, associated JAKs become activated and phosphorylate STAT proteins. Following phosphorylation, STATs translocate to the nucleus and regulate transcription of ISGs which drive activation of immune responses including proinflammatory cytokine production, differentiation, and proliferation of innate and adaptive immune cells, and consequently resulting in systemic hyperinflammation. IL-1α/β, interleukin-1 alpha and beta; ISGs, interferon-signature genes; JAK, janus kinase; STAT, signal transducer and activator of transcription proteins; TNFα, tumour necrosis factor alpha.

Until recently, there were no available treatment options to block type-I IFN signalling pathways; however, better understanding of molecular disease mechanisms guided development of selective janus kinase (JAK) 1/2 inhibitors, baricitinib and ruxolitinib (69) (Figure 3). To date, various JAK inhibitors have been approved as treatment options in inflammatory conditions ranging from rheumatoid arthritis and psoriasis to inflammatory bowel disease (72). Safety and efficacy of baricitinib were evaluated in a recent longitudinal study which demonstrated improvement in quality-of-life scores, growth and development of patients with reduced inflammatory markers, disease activity and ISGs (69). The commonest adverse events were reported as upper respiratory tract infections, gastroenteritis, and viral reactivation with BK virus (69).

### Use of Janus Kinase Inhibition in Children With Interferonopathies at Great Ormond Street Hospital

Herein, novel unpublished data regarding patients diagnosed with interferonopathies and followed-up under Great Ormond Street Hospital (GOSH) paediatric immunology and rheumatology specialities are presented. In our cohort, JAK 1/2 inhibition has been proven to be successful in monogenic and autoimmune interferonopathies. Baricitinib has been used for a median of 9,5 months (range: 5,5 – 27 months). Eleven patients followed by rheumatology (*n* = 10) and immunology (*n* = 1) specialities are reported. Median age was 6 years (range: 2 – 16 years). Seven of 11 patients were diagnosed with monogenic interferonopathies: 2/7 with SAVI had confirmed mutations in *STING1*, 2/7 with AGS (*TREX1* and *SAMHD1* mutations), 3/7 with unclassified interferonopathy. All three unclassified patients had undergone whole exome sequencing that were awaited at the time of writing of this manuscript. The remaining four patients had the following autoimmune interferonopathies: 2/4 systemic lupus erythematosus (SLE), 1/4 juvenile dermatomyositis (JDM), 1/4 autoimmune hepatitis with a detected gain-of-function mutation in STAT1 (Table 2). Physician global assessment (PGA) of the disease activity is a subjective score given by the evaluating clinician and ranges from 0 to 10; 0 indicating well-controlled disease activity. Assessments were carried out by different clinicians, although there has been a remarkable decrease in average PGA from 4.7/10 to 1.3/10 from start of treatment to the last assessment.

**TABLE 2:** Summarised features of our cohort with monogenic and autoimmune interferonopathies treated with selective JAK 1/2 inhibitor baricitinib.

Patient	Age	Sex	Diagnosis	Genotype	Peripheral blood ISG assay	Duration of baricitinib treatment (months)	Current dose	PGA before treatment	PGA at last visit	Comment
Pt 1	13	M	SAVI	*STING1* (p.C206Y)	Abnormal	24	4 mg TDS	6	1	Improved QoL
Pt 2	5	F	Unclassified IFNopathy	Awating WES	Abnormal	27	2 mg TDS	6	2	Improved chilblains, stable neurology and MRI brain scans
Pt 3	8	F	Unclassified IFNopathy	Awating WES	Abnormal	5,5	2 mg TDS	5	2	Significant improvement
Pt 4	2	M	Unclassified IFNopathy	Awating WES	Abnormal	7,5	2 mg BD	4	0	Complete response
Pt 5	8	M	AGS	*SAMHD1* Compound heterozygous (c.400C > T/c.1244A > G)	Abnormal	5,5	2 mg TDS	5	3	Improved chilblains, stable neurology
Pt 6	3	M	SAVI	*STING1* (p.F279L)	Abnormal	11	2 mg TDS	3	1	Post-allo BMT, damage accrued prior to treatment
Pt 7	6	M	JDM	2 VUS *OTULIN* (p.Q115H) *ADA-2 (p.R154C)*	Abnormal	9	2 mg BD	N/A	N/A	Resolution of skin disease, reduction in steroid use, weaning IVIg
Pt 8	4	F	AGS	*TREX1* (p.D73N)	Abnormal	9,5	2 mg TDS	4	1	Reduction in fever and panniculitis episodes, neurology stable
Pt 9	16	F	SLE	N/A	Abnormal	11	4 mg BD	3	0.5	DLQI at start: 15/30 DLQI last visit: 0/30
Pt 10	15	F	SLE	N/A	Abnormal	6,5	4 mg BD	N/A	N/A	Unclear, compliance issues
Pt 11	3	F	Autoimmune hepatitis	STAT1 Gof	Not done	10	2 mg am/pm 4 mg lunch	7	2	Significant improvement

*ADA-2, Adenosine-deaminase-2; AGS, Aicardi-Goutières Syndrome; Allo-BMT, allogeneic bone marrow transplantation; BD, bis in die (twice a day); DLQI, dermatology life quality index; Gof, gain-of-function; IFNopathy, interferonopathy; ISG, interferon signature gene; JDM, juvenile dermatomyositis; N/A, not applicable; OTULIN, OTU deubiquitinase with linear linkage specificity; pt, patient; SAMHD1, SAM and HD domain containing deoxynucleoside triphosphate triphosphohydrolase 1; SAVI, STING-associated vasculopathy of infancy; SLE, systemic lupus erythematosus; STAT1, signal transducer and activator of transcription protein 1; STING1, stimulator of interferon response CGAMP interactor 1; QoL, quality of life; TDS, ter in die (three times a day); TREX1, Three prime repair exonuclease 1; VUS, variant of uncertain significance; WES, whole exome sequencing.*

**FIGURE 4:**
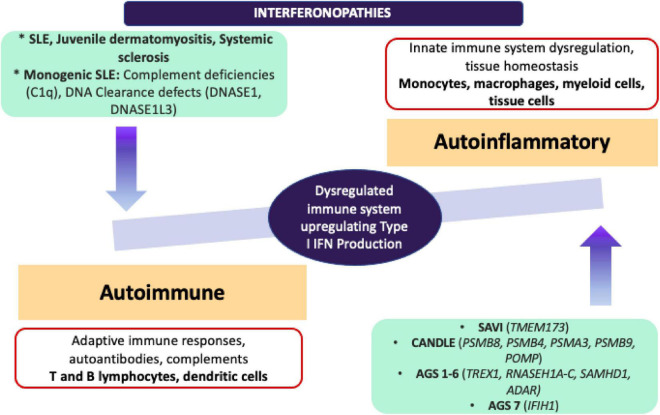
Adapted from Kim et al. (68) comparison of autoinflammatory and autoimmune interferonopathies. Monogenic autoinflammatory interferonopathies that are demonstrated on the right end, are currently defined as CANDLE/PRAAS, SAVI and AGS, and driven by the mutations in proteasome complexes resulting in dysregulation in innate immune responses and increased transcription of IFN signature genes without immune complex deposition. On the left end, monogenic SLE, JDM and SSc are shown under autoimmune interferonopathies, since fundamentally adaptive immune system drives the underlying autoimmune pathology with production of autoantibodies, deposition of immune complexes and activation of T and B lymphocyte subsets. Pathogenic immune complexes can trigger IFN production in these conditions through activation of B lymphocytes and dendritic cells. ADAR, Adenosine deaminase RNA specific; AGS, Aicardi-Goutières Syndrome; CANDLE, Chronic Atypical Neutrophilic Dermatosis with Lipodystrophy and Elevated temperature; C1q, complement 1q; DNASE, deoxyribonuclease; IFIH1, Interferon induced with helicase C domain 1; IFN, interferon; JDM, juvenile dermatomyositis; POMP, Proteasome maturation protein; PSMA3, Proteasome 20S subunit alpha 3; PSMB 4/8/9, Proteasome 20S subunit beta 4/8/9; RNASE H1A-C, ribonuclease H1A-C; SAMHD1, SAM and HD domain containing deoxynucleoside triphosphate triphosphohydrolase 1; SLE, systemic lupus erythematosus; TMEM173, transmembrane protein 173; TREX1, three prime repair exonuclease 1.

It would be important to emphasise that in addition to monogenic interferonopathies, ISGs have been found upregulated in other autoimmune conditions such as SLE, JDM, and systemic sclerosis. Further scrutiny of the molecular pathogenesis in type I IFN signature has not only led to the development of novel targetted-therapeutic options for Mendelian interferonopathies and for other autoimmune diseases, but also has increased our appreciation of the continuum between the autoinflammatory and autoimmune conditions (73) (Figure 4).

## Conclusion

The list of monogenic SAIDs has been ever-growing with the advances in molecular genetic testing and increased awareness amongst physicians. Although the notion that autoinflammatory conditions are driven by innate immune dysregulation is generally true, identification of novel mutations in genes causing overlap with autoinflammation, immunodeficiency, and/or autoimmunity has strongly indicated the crosstalk between the adaptive and innate immune systems.

**FIGURE 5:**
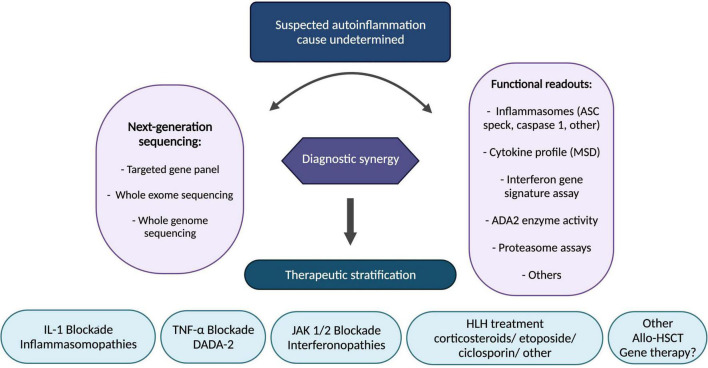
Stratified therapeutic approach for autoinflammation. Functional readouts listed are non-exhaustive examples; other functional readouts pertinent to any genetic findings include STAT signalling (interferon pathway); ADA-2 enzyme activity; leucocyte intracellular MVK enzyme activity; peripheral blood RNA interferon signature, amongst others. ADA-2, adenosine-deaminase 2; Allo-HSCT, allogeneic haematopoietic stem cell transplantation; ASC, apoptosis associated speck-like protein containing a CARD; DADA2, deficiency of adenosine deaminase 2; HLH, hemophagocytic lymphohistiocytosis; IL-1, interleukin-1; JAK, janus kinase; MSD, meso scale discovery; TNF, tumour necrosis factor.

Elucidation of the underlying innate immune mechanisms with translational and reverse-translational research has facilitated precision medicine in this space, particularly resulting in therapeutic stratification to IL-1blockade (inflammasomopathies), anti-TNF (DADA2), and JAK 1/2 inhibitors (interferonopathies). Ongoing challenges as more and more patients have access to next generation genetic sequencing include how to interpret novel genetic variants; functional assays to probe the pathogenicity of genetic variants are increasingly necessary in routine clinical practice. Gene therapies are on the horizon for autoinflammation: *ex vivo* lentiviral transduction of haematopoietic stem cells; gene editing; and gene silencing are all likely to impact patient care in the coming decade, and offer hope especially for treatment-resistant/inadequately controlled cases. In the meantime, we present a summary of our therapeutic stratification strategy for autoinflammation including fulminant inflammation caused by monogenic diseases associated with haemophagocytic lymphohistiocytosis and related entities (Figure 5).

## Data Availability Statement

The original contributions presented in the study are included in the article/supplementary material, further inquiries can be directed to the corresponding author.

## Ethics Statement

This study was approved in full by the NRES Committee London – Bloomsbury, ethics number 08/H0713/82. Written informed consent was obtained from the minor(s)’ legal guardian/next of kin for the publication of any potentially identifiable images or data included in this article.

## Author Contributions

OK drafted the manuscript. AP, KW, and OK designed the figures and tables. DE and PB revised and finalised the manuscript. All authors contributed to the article and approved the submitted version.

## Author Disclaimer

The views expressed are those of the author(s) and not necessarily those of the NHS, the NIHR, or the Department of Health.

## Conflict of Interest

The authors declare that the research was conducted in the absence of any commercial or financial relationships that could be construed as a potential conflict of interest.

## Publisher’s Note

All claims expressed in this article are solely those of the authors and do not necessarily represent those of their affiliated organizations, or those of the publisher, the editors and the reviewers. Any product that may be evaluated in this article, or claim that may be made by its manufacturer, is not guaranteed or endorsed by the publisher.

## Footnotes

1
http://fmf.igh.cnrs.fr/ISSAID/infevers

2
http://fmf.igh.cnrs.fr/infevers/
